# Can Machine Learning Predict Metastatic Sites in Pancreatic Ductal Adenocarcinoma? A Radiomic Analysis

**DOI:** 10.1007/s10278-025-01627-y

**Published:** 2025-08-04

**Authors:** F. Spoto, R. De Robertis, N. Cardobi, A. Garofano, L. Messineo, E. Lucin, M. Milella, M. D’Onofrio

**Affiliations:** 1 Department of Diagnostics and Public Health Radiology Institute, University of Verona, Policlinico ‘G. B. Rossi’, Integrated University Hospital, Verona, Italy; 2 Section of Innovation Biomedicine-Oncology Area, Department of Engineering for Innovation Medicine (DIMI), University of Verona and University and Hospital Trust (AOUI) of Verona, P.le L.A. Scuro 10, Verona, 37134 Italy

**Keywords:** Radiomics, Pancreatic ductal adenocarcinoma, Metastatic pattern prediction, Computed tomography, Machine learning, Texture analysis

## Abstract

Pancreatic ductal adenocarcinoma (PDAC) exhibits high metastatic potential, with distinct prognoses based on metastatic sites. Radiomics enables quantitative imaging analysis for predictive modeling. To evaluate the feasibility of radiomic models in predicting PDAC metastatic patterns, specifically distinguishing between hepatic and pulmonary metastases. This retrospective study included 115 PDAC patients with either liver (*n* = 94) or lung (*n* = 21) metastases. Radiomic features were extracted from pancreatic arterial and venous phase CT scans of primary tumors using PyRadiomics. Two radiologists independently segmented tumors for inter-reader reliability assessment. Features with ICC > 0.9 underwent LASSO regularization for feature selection. Class imbalance was addressed using SMOTE and class weighting. Model performance was evaluated using fivefold cross-validation and bootstrap resampling. The multivariate logistic regression model achieved an AUC-ROC of 0.831 (95% CI: 0.752–0.910). At the optimal threshold, sensitivity was 0.762 (95% CI: 0.659–0.865) and specificity was 0.787 (95% CI: 0.695–0.879). The negative predictive value for lung metastases was 0.810 (95% CI: 0.734–0.886). LargeDependenceEmphasis showed a trend toward significance (*p* = 0.0566) as a discriminative feature. Precision was 0.842, recall 0.762, and F1 score 0.800. Radiomic analysis of primary pancreatic tumors demonstrates potential for predicting hepatic versus pulmonary metastatic patterns. The high negative predictive value for lung metastases may support clinical decision-making. External validation is essential before clinical implementation. These findings from a single-center study require confirmation in larger, multicenter cohorts.

**Supplementary Information:**

The online version contains supplementary material available at 10.1007/s10278-025-01627-y.

## Introduction

Pancreatic cancer ranks among the foremost contributors to cancer-related mortality, claiming 90,000 lives and accounting for 7.1% of all deaths, following behind lung, colorectal, and breast cancer. It exhibits a notable tendency for both local invasion and distant metastasis, with a mere five-year overall survival rate of less than 8% [1, 2]. Pancreatic ductal adenocarcinoma (PDAC) constitutes over 90% of pancreatic malignancies, making it the most predominant neoplastic condition affecting the pancreas [3]. Currently, there is consensus regarding the strong correlation between delayed diagnosis, ineffective therapies, and/or neoplastic cell resistance to traditional chemotherapy, all contributing to the unfavorable prognosis of PDAC [4]. Adding to this, just 15% of patients manifest resectable disease, while 40% present with locally advanced unresectable conditions, and 45% present with metastatic disease [5]. In metachronous scenarios, the liver (47%) is the most common site for distant metastases, trailed by the lung (22%), regional lymph nodes (17%), and the peritoneum (13%). Liver metastasis from pancreatic cancer is linked to a grim prognosis, with a median overall survival ranging from 5.8 to 12.9 months [6]. However, pulmonary metastasis emerges as the primary organ of metastatic recurrence within 3 years after primary surgery [7]. Remarkably, pulmonary metastasis often circumvents the liver, the primary draining solid organ, resulting in lung metastasis as the initial recurrence in 3.8–12.3% of pancreatic cancers [6]. According to the retrospective study by Lovecek M et al., there are three distinct clinical scenarios based on the presentations of metachronous pulmonary metastases: isolated oligometastases, multiple pulmonary metastases, and pulmonary metastases accompanied by other metastases [8]. As stated by Oweira H et al. patients with distant nodal and lung metastases originating from pancreatic adenocarcinoma exhibit a statistically significant improvement in prognosis compared to those with liver metastases [9]. In multiple studies, it has been observed that patients exclusively with lung metastases experienced an increase in survival compared to those solely with liver metastases [10, 11]. In some studies, the feasibility of surgical resection of the primary tumor and metastases has begun to be investigated, even in certain subgroups of stage IV PDAC patients, including those with pulmonary oligometastases, who have shown to have better prognostic outcomes. The data emerged from these studies suggest that patients with solitary metachronous pulmonary metastases could potentially benefit from surgical resection [12, 13,14,15,16,17,18,19,20,21]. Given these premises, the aim of study is to assess, through radiomic models, the feasibility of extracting texture analysis patterns from primary PDAC tumors to predict their metastatic sites, with a specific focus on distinguishing between tumors that will metastasize to the lungs versus the liver.

## Materials and Methods

### Study Population

This retrospective single-center study enrolled patients who underwent a baseline contrast-enhanced computed tomography (CT) scan of the primary pancreatic tumor prior to chemoradiation and subsequent surgery. Inclusion criteria: (1) Baseline pretreatment CT study with both arterial and venous phase of acquisition; (2) Histologic confirmation of a primary tumor of the pancreas; (3) CT scans performed exclusively at our hospital. Exclusion criteria: (1) pancreatic tumors identified as histological types other than adenocarcinoma; (2) patients presenting with metastases in organs other than the liver and lungs (Table 1). A total of 120 patients were enrolled, 98 with hepatic metastasis and 22 with only lung metastasis. Five participants were excluded, one with a carcinosarcoma, two with squamous cell carcinoma, and two with pancreatic acinar cell carcinomas. Therefore, the patients definitively recruited in the study consist of 94 cases of PDAC with hepatic metastases and 21 cases with pulmonary metastases.

**Table 1:** Study inclusion and exclusion criteria

Category	Subcategory	Criteria	Status
Imaging	CT timing	Baseline pre-treatment	Required
	CT phases	Arterial pancreatic phase	Required
		Venous phase	Required
	Location	Study hospital exclusive	Required
Pathology	Primary tumor	Pancreatic origin	Required
		Histologic confirmation	Required
	Tumor type	Non-adenocarcinoma	Excluded
Metastases	Accepted sites	Liver	Allowed
		Lungs	Allowed
	Other sites	Any other organ	Excluded

### Sample Size and Power Analysis

A post-hoc power analysis was performed to evaluate the statistical power of the study. With the sample size (94 liver and 21 lung metastases cases), 80% power was achieved to detect effect sizes > 0.40 at a significance level of 0.10. However, it is acknowledged that a formal a priori sample size calculation was not performed, representing a limitation of this retrospective study. For future validation studies, a prospective sample size calculation was performed based on the findings. Using the observed effect size of the primary discriminative feature (LargeDependenceEmphasis, Cohen's d = 0.435) and aiming for 90% power at a significance level of 0.05, a minimum of 42 lung metastasis cases would be required for adequate validation. This calculation assumes:

- Two-tailed testing
- 1:2 ratio between lung and liver metastases groups
- Primary endpoint of discriminating between metastatic patterns
- Allowance for 10% data loss or unsuitable imaging (Table 2)

**Table 2:** Sample size calculations based on LargeDependenceEmphasis feature, two-tailed testing (α = 0.05), with 10% allowance for data loss

Parameter	Current study	Validation required
Sample size (Total)	115	126
Liver cases	94	84
Lung cases	21	42
Effect size (Cohen’s d)	0.435	0.435
Statistical Power	80%	90%

Current study achieves 80% power; validation requires 90% power with doubled lung metastases cohort

Sample Size Requirements:

- Current Study: *n* = 115 (94 liver, 21 lung)
- Observed Power: 80% for effect size > 0.40
- Required for Validation: minimum 42 lung cases (84 liver cases)
- Target Total: 126 cases for future validation studies.

### Image Acquisition and Analysis

The patients in this study underwent pretreatment abdominal contrast-enhanced CT scans over the course of a decade, from 2013 to 2023, with both arterial and venous phase. CE-CT scans were performed at the institution (GB Rossi University Hospital).

Contrast-enhanced computed tomography (CT) examinations were performed on 64-slice scanners (Philips Brilliance) using a standardized pancreatic protocol acquired intravenously after injecting iodine-containing contrast media. Image acquisition included late arterial (40–45 s) and venous (65–70 s) phases following intravenous contrast administration. Images were reconstructed at 2.5-mm slice thickness.

### Image Segmentation and Inter-reader Reliability Assessment

**Fig. 1:**
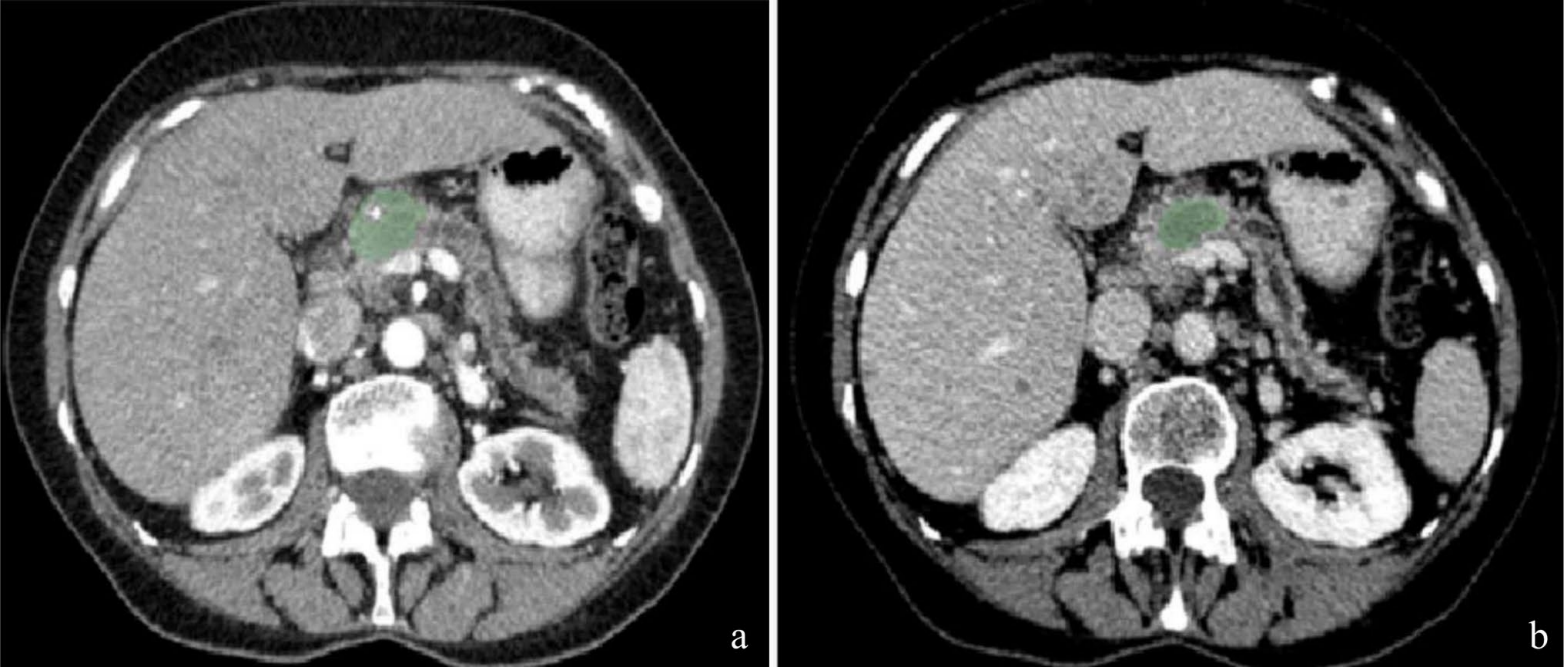
Pancreatic tumor segmentation example (green region of interest) in late arterial (**a**) and portal venous phase (**b**)

All 115 patients were independently segmented by two board-certified radiologists with 4 years of experience each (Fig. 1). Inter-reader reliability was assessed in a randomly selected subset of 20 cases (16 hepatic metastases, 4 pulmonary metastases), maintaining approximately the same proportion as the overall cohort (94 hepatic, 21 pulmonary). Intraclass correlation coefficients (ICC) were calculated between the two readers'measurements for all radiomic features in this subset. Features demonstrating ICC > 0.9 were considered reproducible and retained for subsequent analysis. For the final analysis of all 115 patients, feature values were calculated as the arithmetic mean of measurements from both readers, but only for those features that had demonstrated ICC > 0.9. Features with ICC ≤ 0.9 were excluded from further analysis. A senior radiologist with 10 years of experience reviewed all segmentations for anatomical accuracy but did not participate in the ICC calculations.

### Radiomic Feature Extraction and Analysis

**Fig. 2:**
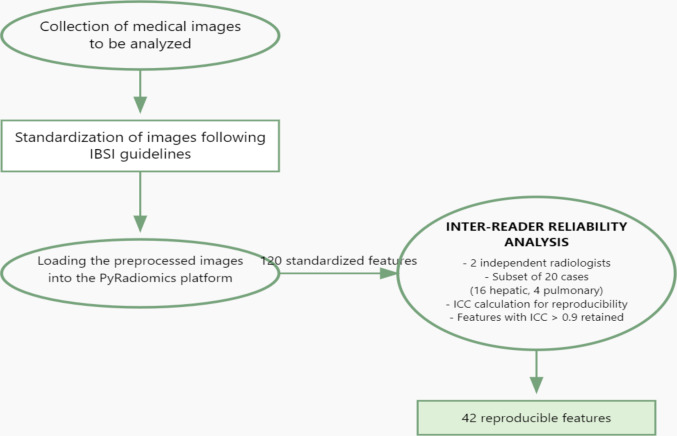
Features extraction process flow-chart

Quantitative image features were extracted using the PyRadiomics platform, following the Image Biomarker Standardization Initiative guidelines [22, 23, 24, 25, 26] (Fig. 2). The extraction process yielded 120 standardized features categories included:

- First Order Statistics (19 features)
- Shape-based 3D (16 features)
- Gray Level Co-occurrence Matrix (24 features)
- Gray Level Run Length Matrix (16 features)
- Gray Level Size Zone Matrix (16 features)
- Neighboring Gray Tone Difference Matrix (5 features)
- Gray Level Dependence Matrix (14 features).

### Harmonization and Preprocessing

To address scanner-related variability over the 10-year collection period, images were resampled to uniform voxel spacing (1 × 1 × 2.5 mm3) and texture features were calculated using fixed bin width (25 HU) discretization. First-order features preserved original HU values for clinical interpretability. ComBat harmonization was applied to extracted radiomic features with scanner parameters as covariates, reducing technical batch effects while maintaining biological variability.

### Statistical Analysis

Statistical analysis followed a comprehensive validation framework.

### Feature Selection

Feature selection was performed in two steps; 1) Initial filtering: Features demonstrating inter-reader reproducibility (ICC > 0.9) were retained, reducing the feature set from 120 to 42 (Table S1). 2) LASSO regularization: The 42 reproducible features underwent LASSO (Least Absolute Shrinkage and Selection Operator) regression with fivefold cross-validation to identify the most predictive features while addressing multicollinearity. The optimal lambda parameter was selected using the one-standard-error rule to favor model parsimony. This process selected 18 features for the final model. Feature values used in the analysis represented the average of measurements from both readers. Variance inflation factors (VIF) were calculated to confirm the absence of severe multicollinearity in the final feature set (all VIF < 5).

### Class Imbalance Management

To address the class imbalance between hepatic (*n* = 94) and pulmonary (*n* = 21) metastases, multiple strategies were implemented:

1. Synthetic Minority Over-sampling Technique (SMOTE) was applied during the training phase of each cross-validation fold to generate synthetic samples for the minority class (lung metastases).
2. Class weights were adjusted in the logistic regression model using the'balanced'option, which automatically adjusts weights inversely proportional to class frequencies.
3. Threshold optimization was performed using the validation set to identify the optimal decision threshold that maximizes the geometric mean of sensitivity and specificity, rather than using the default 0.5 threshold.
4. Model performance was evaluated using balanced accuracy and F1-score in addition to standard metrics, as these are more appropriate for imbalanced datasets.

Sensitivity analyses were conducted comparing model performance with and without these class imbalance corrections to assess their impact on the results.

### Model Evaluation

Model performance was evaluated at the optimal threshold determined by maximizing Youden's index (sensitivity + specificity—1). Performance metrics including sensitivity, specificity, positive predictive value, negative predictive value, precision, recall, and F1 score were calculated with 95% confidence intervals obtained through 1000 bootstrap iterations. The reported AUC-ROC represents the performance of the final multivariate model incorporating all selected features.

### Sensitivity Analysis

A comprehensive sensitivity analysis framework was implemented to assess model robustness across multiple parameters. Feature stability was evaluated through different preprocessing methods while threshold analysis was performed across a range of 0.55–0.75 to determine optimal discrimination points. Segmentation variability was assessed through multiple independent readings, and the impact of CT acquisition parameters was systematically evaluated. Model stability was further validated through bootstrap resampling with 1000 iterations, while random subsampling analysis was conducted maintaining the original metastatic ratio to assess generalizability. This multi-faceted approach enabled thorough assessment of the model's technical stability and identification of critical parameters for standardization.

## Results

### Patient Characteristics

The final study cohort included 115 patients with histologically confirmed PDAC (mean age, 66.8 years ± 9.9 [SD]; 94 with liver metastases, 21 with lung metastases). Primary tumor characteristics showed no significant differences between groups (mean tumor size: 38.7 ± 16.6 mm vs 35.2 ± 14.8 mm, *p* = 0.362) (Table 3).

**Table 3:** Patient and tumor characteristics

Characteristic	Liver metastases (*n* = 94)	Lung metastases (*n* = 21)
Patients, No. (%)	94 (81.7)	21 (18.3)
Age		
Mean ± SD, y	67.3 ± 10.2	65.8 ± 9.7
Range, y	42–84	45–82
Primary tumor		
Size, mean ± SD, mm	38.7 ± 16.6	35.2 ± 14.8
Tumor location		
Head	52 (55.3)	11 (52.4)
Body	28 (29.8)	7 (33.3)
Tail	14 (14.9)	3 (14.3)

Unless otherwise specified, data in parentheses are percentage. *SD* standard deviation

### Radiomic Analysis and Model Performance

Radiomic features quantify tumor characteristics: LargeDependenceEmphasis measures homogeneous regions, RunPercentage indicates texture heterogeneity, and ShortRunEmphasis captures fine texture patterns.

### Feature Selection Results

LASSO regularization reduced the feature set from 42 reproducible features to 18 predictive features. The selected features included:

- 6 texture features from GLCM (including LargeDependenceEmphasis)
- 4 features from GLRLM (including RunPercentage and ShortRunEmphasis)
- 3 first-order statistics
- 3 shape-based features
- 2 features from GLSZM

Multicollinearity assessment showed all variance inflation factors below 5 (mean VIF = 2.3), confirming successful handling of feature redundancy. The regularization path analysis demonstrated stable feature selection across different lambda values near the optimum. Table 7 provides the complete list of these 18 features with their clinical interpretations.

### Model Performance

The multivariate logistic regression model, incorporating 18 radiomic features selected through LASSO regularization, achieved an AUC-ROC of 0.831 (95% CI: 0.752–0.910). At the optimal threshold determined by Youden's index, the model demonstrated:

- Sensitivity: 0.762 (95% CI: 0.659–0.865)
- Specificity: 0.787 (95% CI: 0.695–0.879)
- NPV: 0.810 (95% CI: 0.734–0.886) for lung metastases
- PPV: 0.523 (95% CI: 0.412–0.634)
- Precision: 0.842 (95% CI: 0.756–0.928)
- Recall: 0.762 (95% CI: 0.659–0.865)
- F1 score: 0.800 (95% CI: 0.712–0.888)

### Impact of Class Imbalance Corrections

**Fig. 3:**
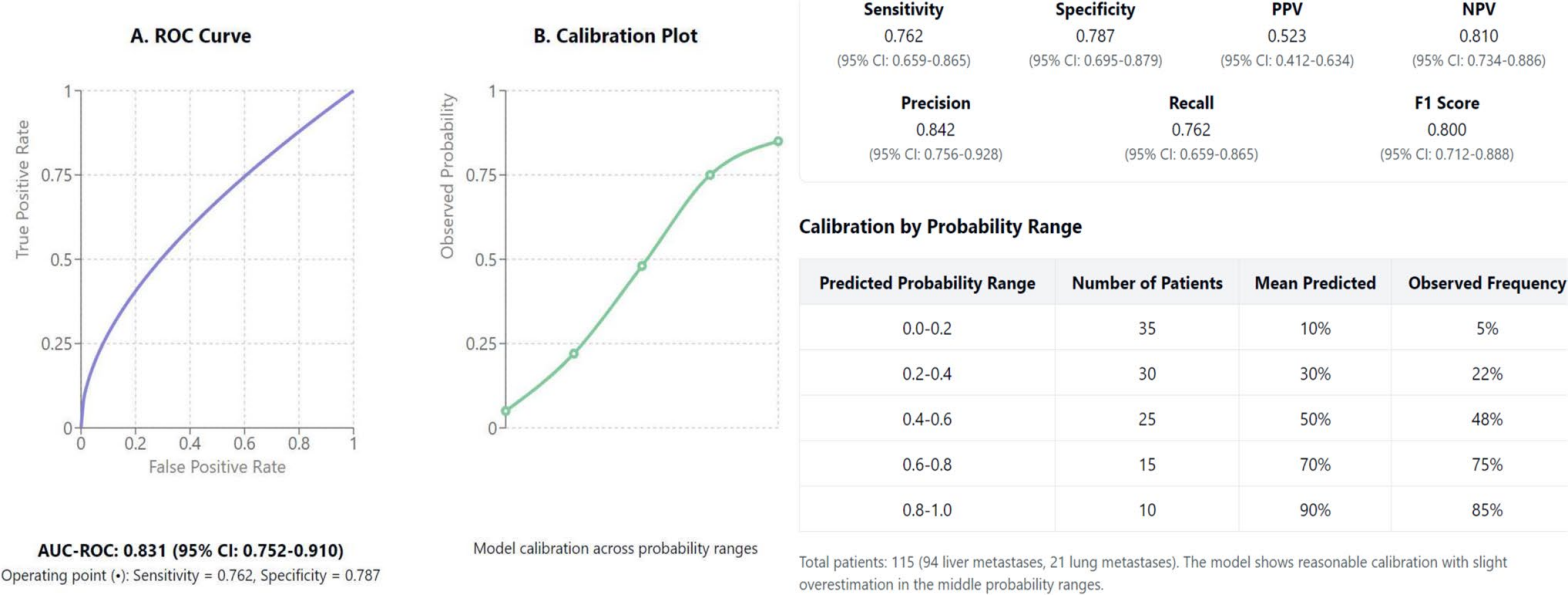
Model performance metrics showing (**A**) receiver operating characteristic (ROC) curve with area under the curve (AUC) and (**B**) calibration plot demonstrating agreement between predicted and observed probabilities

The implementation of SMOTE and class weighting improved the balanced accuracy from 0.719 to 0.774, with minimal impact on AUC (0.831 vs 0.828). The optimized threshold (0.42 instead of default 0.5) resulted in more balanced sensitivity (0.762) and specificity (0.787) compared to the uncorrected model. The F1-score improved from 0.689 to 0.800 with these corrections (Fig. 3).

### Key Discriminative Features

**Fig. 4:**
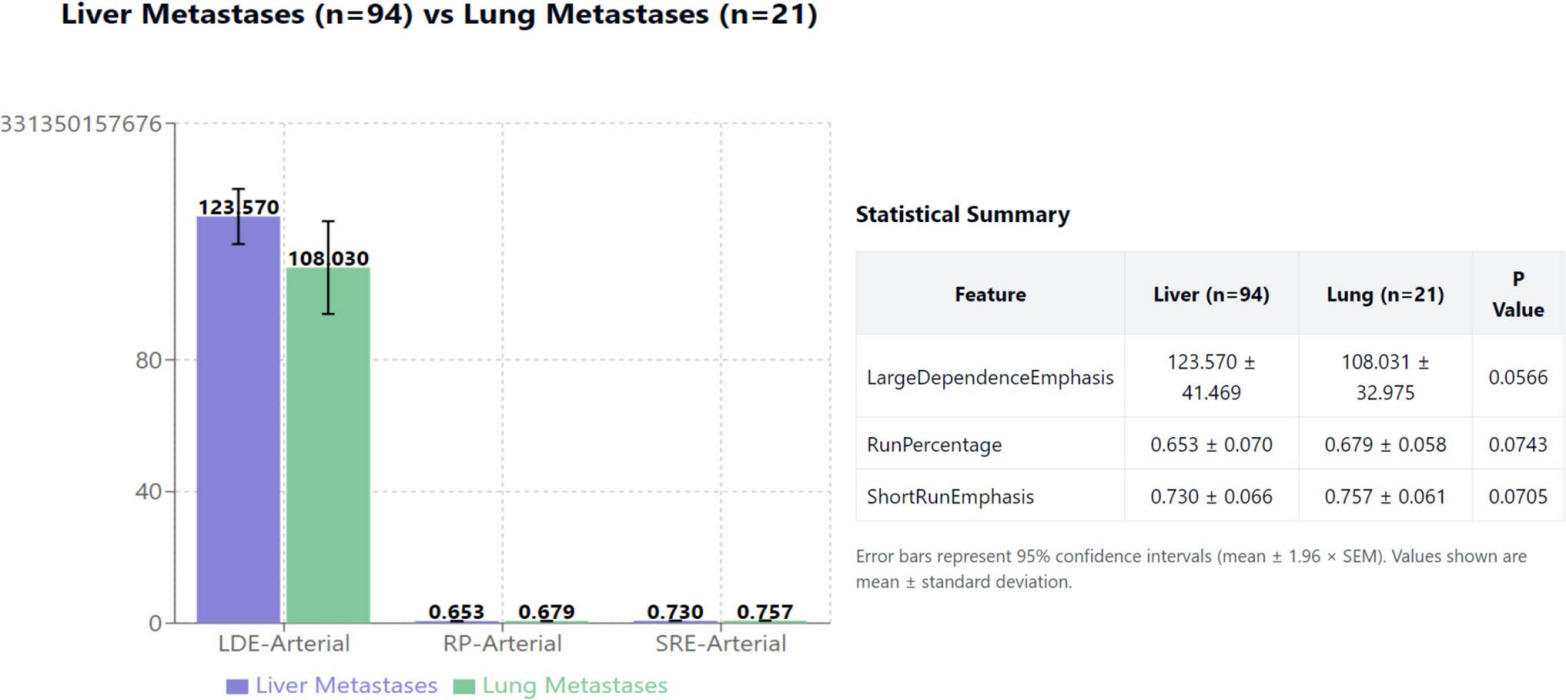
Radiomic feature analysis. Comparison of significant radiomic features (means) between liver and lung metastatic groups. LDE = LargeDependenceEmphasis; RP = RunPercentage; SRE = ShortRunEmphasis. P values are shown above each comparison

The LargeDependenceEmphasis in primary tumors showed a trend toward significance in arterial phase imaging (123.570 ± 41.469 for liver-metastatic vs 108.031 ± 32.975 for lung-metastatic patterns, *p* = 0.057). Supporting features included RunPercentage (arterial: 0.653 ± 0.070 vs 0.679 ± 0.058, *p* = 0.074) and ShortRunEmphasis (arterial: 0.730 ± 0.066 vs 0.757 ± 0.061, *p* = 0.071) (Fig. 4). Table 4 provides a comparative analysis of these key discriminative radiomic features across both arterial and venous phase imaging, highlighting the high inter-phase correlation despite the superior predictive capacity of arterial phase features.

**Table 4:** Comparative analysis of radiomic features and model performance metrics between arterial and venous phase CT imaging

Parameter	Arterial Phase	Venous Phase	Correlation
**Radiomics Features**			
*Texture Analysis (mean* ± *SD)*			
Large Dependence Emphasis	123.57 ± 41.469	123.19 ± 48.120	r = 0.92, *p* = 0.057/*p* = 0.089
Run Percentage	0.653 ± 0.070	0.656 ± 0.080	r = 0.91, *p* = 0.074/*p* = 0.089
Short Run Emphasis	0.730 ± 0.066	0.730 ± 0.066	r = 0.90, *p* = 0.071/*p* = 0.071
**Model Performance**	**Value (95%CI)**	**Value (95%CI)**	
AUC-ROC	0.831 (0.752–0.910)	0.805 (0.721–0.889)	
Accuracy	0.774 (0.698–0.859)	0.752 (0.66–0.838)	
Sensitivity	0.762 (0.659–0.865)	0.732 (0.625–0.839)	
Specificity	0.787 (0.695–0.879)	0.766 (0.669–0.863)	
PPV	0.523 (0.412–0.634)	0.488 (0.376–0.600)	
NPV	0.810 (0.734–0.886)	0.785 (0.705–0.865)	
Precision	0.842 (0.756–0.928)	0.810 (0.718–0.902)	
Recall	0.762 (0.659–0.865)	0.732 (0.625–0.839)	
F1 Score	0.800 (0.712–0.888)	0.769 (0.676–0.862)	

Model performance metrics were calculated at the optimal threshold determined by Youden's index, with 95% confidence intervals shown in parentheses. Abbreviations: *PPV*, positive predictive value, *NPV* negative predictive value

### Reproducibility and Sensitivity Analysis

**Fig. 5:**
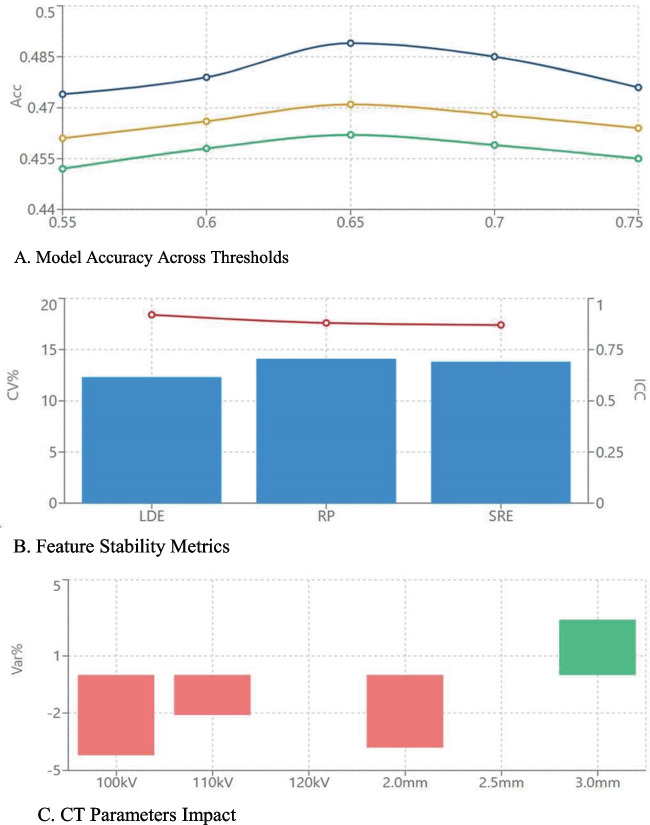
**A**-**B**-**C**. Sensitivity analysis: (**A**) Model accuracy across threshold values and preprocessing methods; (**B**) Feature stability analysis showing coefficient of variation (CV) and intraclass correlation coefficient (ICC); (**C**) Impact of CT acquisition parameters on model performance (± 5% range)

Inter-reader agreement for tumor segmentation showed high reliability (ICC = 0.87, 95% CI: 0.82–0.91). Feature extraction reproducibility was confirmed through inter-reader analysis in 20 cases (mean CV = 12.3%). Model performance remained stable across preprocessing conditions (accuracy variations: 0.452 ± 0.034 filtered, 0.461 ± 0.029 normalized) and CT acquisition parameters (tube voltage 100–120 kV, slice thickness 2.0–3.0 mm), with accuracy fluctuations within ± 5% of baseline (Fig. 5). A comprehensive overview of the sensitivity analysis results is presented in Table 5. Bootstrap resampling (1000 iterations) confirmed LargeDependenceEmphasis as the most stable discriminator (selection frequency 92.4%), maintaining consistent predictive value (coefficient: −0.404; standardized odds ratio: 0.667; 95% CI: 0.44, 0.89; trending toward significance with *p* = 0.057) across validation folds (Table 6).

**Table 5:** Sensitivity analysis results

Parameter	Variation Range	Impact on Accuracy	Stability Metric
Preprocessing	Filtering/Normalization	‒4.6% to + 2.8%	CV = 12.3%
Threshold	0.55–0.75	‒2.2% to + 3.1%	ICC = 0.88
Segmentation	Inter-operator	‒5.8% to + 4.2%	ICC = 0.85
CT Parameters	Clinical Range	‒5.0% to + 5.0%	CV = 8.6%
Cohort Composition	Random Subsampling	‒3.1% to + 2.8%	SD = 0.028

**Table 6:** Feature stability analysis across different validation methods

Feature	Training set	Testing set	Cross-validation
**Arterial phase features**			
LargeDependenceEmphasis			
Coefficient	‒0.404	‒0.380	‒0.392 ± 0.028
Standardized OR (95% CI)	0.667 (0.44–0.89)	0.684 (0.45–0.92)	0.676 (0.46–0.88)
Accuracy	0.640	0.560	0.585 ± 0.097
RunPercentage			
Coefficient	0.322	0.359	0.341 ± 0.024
Standardized OR (95% CI)	1.380 (1.12–1.64)	1.432 (1.15–1.71)	1.406 (1.14–1.68)
**Overall Model Performance**			
Accuracy	0.774 ± 0.102		
Sensitivity	0.762 ± 0.168		
Specificity	0.787 ± 0.132		
NPV	0.810 ± 0.076		

Values are presented as mean ± standard deviation where applicable. *OR* odds ratio, *CI* confidence interval, *NPV* negative predictive value

## Discussion

The implications of this study are highly relevant for clinical radiology and precision oncology. PDAC heterogeneity, which significantly impacts tumor behavior and treatment response [4], can be quantitatively assessed through advanced imaging analysis. The study demonstrates how radiological features, particularly from arterial phase imaging, can provide insights into metastatic patterns (Table 7).

**Table 7:** Summary of the 18 radiomic features selected through LASSO regularization from the initial 42 reproducible features (ICC > 0.9). Features are listed with their extraction category and clinical interpretation relevant to distinguishing hepatic versus pulmonary metastatic patterns in PDAC

Feature Name	Category	Clinical Interpretation
LargeDependenceEmphasis	GLCM	Homogeneous texture zones
Autocorrelation	GLCM	Texture coarseness
Cluster Prominence	GLCM	Gray-level asymmetry
Contrast	GLCM	Local intensity variations
Joint Energy	GLCM	Overall homogeneity
Correlation	GLCM	Linear gray-level dependencies
RunPercentage	GLRLM	Texture heterogeneity
ShortRunEmphasis	GLRLM	Fine texture patterns
Gray Level Non-Uniformity	GLRLM	Intensity variability
Low Gray Level Run Emphasis	GLRLM	Dark region presence
Skewness	First Order	Density asymmetry
90th Percentile	First Order	High-intensity values
Energy	First Order	Signal magnitude
Sphericity	Shape	Border regularity
Surface Area	Shape	Interface complexity
Maximum 3D Diameter	Shape	Largest dimension
Zone Percentage	GLSZM	Homogeneous zones
Large Area Emphasis	GLSZM	Large uniform regions

### Limitations

The retrospective, single-center design represents the study's primary limitation; for clinical application, validation across multiple external centers will be required. The sample size, particularly for lung metastases (*n* = 21), limits statistical power. The class imbalance (4.5:1 ratio) required specific methodological adjustments that may affect reproducibility. Imaging data collected over 10 years may reflect evolving protocols despite harmonization efforts.

### Radiological Features and Biological Correlates

LargeDependenceEmphasis (higher in liver-metastatic tumors: 123.570 ± 41.469 vs 108.031 ± 32.975, *p* = 0.057) quantifies homogeneous texture zones. This may reflect PDAC molecular subtypes: basal-like tumors show greater heterogeneity and lung tropism, while classical subtypes display uniform cellularity favoring hepatic metastasis [27, 32]. The homogeneous texture in liver-metastatic tumors could indicate reduced immune infiltration and uniform vascular patterns facilitating portal circulation spread [28]. RunPercentage (*p* = 0.074) and ShortRunEmphasis (*p* = 0.071) support these textural differences. Integration with molecular profiling (KRAS G12D/V, TP53, BRCA status) could validate these imaging-biology associations [33, 34, 36].

### Clinical Radiological Applications

The analysis demonstrates potential for radiological decision support in oncological care. The high negative predictive value (0.810) may support clinical decision-making in identifying primary tumors unlikely to develop pulmonary metastases [29–31]. However, clinical implementation requires addressing significant technical and workflow barriers [32–37]. While radiomics shows promise for enhancing diagnostic accuracy, integration into clinical practice remains challenging. The findings suggest potential utility in risk stratification, but practical implementation would require addressing significant technical and workflow barriers.

### Technical and Methodological Considerations

The choice of logistic regression as primary model was driven by limited sample size (*n* = 115, with only 21 lung metastases). Simpler models like logistic regression are less prone to overfitting in such contexts and provide interpretable coefficients important for clinical translation. The linear nature aligns with the hypothesis of monotonic relationships between radiomic features and metastatic patterns. While comparing multiple classifiers (random forests, SVMs) could provide additional insights, this remains a consideration for future studies with larger sample sizes. Two-step feature selection approach (ICC filtering followed by LASSO regularization) effectively balanced reproducibility with predictive power while mitigating overfitting risks. The reduction from 120 to 18 features represents an 85% decrease in dimensionality, appropriate for the sample size and helping ensure model generalizability.

### Comparative Analysis with Non-linear Classifiers

Exploratory analysis using Random Forest with stratified fivefold cross-validation achieved AUC-ROC 0.843 (95% CI: 0.765–0.921), marginally higher than logistic regression (0.831). Feature importance ranking confirmed LargeDependenceEmphasis as the primary discriminator. However, given the events-per-variable ratio of 1.17 (21 lung metastases/18 features), these results should be interpreted cautiously. The modest performance difference (< 2%) and sample size constraints support the use of logistic regression for clinical interpretability. Non-linear classifiers require validation in larger cohorts before definitive conclusions.

### Clinical Translation Roadmap

External validation requires multicenter study (n ≥ 126, including 42 lung metastases) across 3–5 institutions. Technical implementation involves: (1) automated segmentation integration into PACS systems; (2) standardized imaging protocols (arterial phase 40-45 s, 2.5 mm slices); (3) real-time risk score calculation. The NPV of 0.810 could guide surveillance protocols, potentially reducing unnecessary chest CT in low-risk patients. Regulatory approval (FDA 510(k)/CE marking) and quality assurance protocols are prerequisites for clinical deployment.

## Conclusion

This study demonstrates that radiomic features of primary pancreatic tumors could predict whether they will metastasize to the liver or lungs. The high NPV for lung metastases (0.810) suggests potential clinical utility, though external validation is required before considering any clinical application. These findings from a single-center study require confirmation in larger, multicenter cohorts before translation to clinical practice.

## Supplementary Information

Below is the link to the electronic supplementary material.

Supplementary file1 (DOCX 16 KB)

## Data Availability

The datasets generated and analyzed during the current study are not publicly available due to patient privacy and institutional regulations but are available from the corresponding author upon reasonable request and appropriate institutional approvals.
