# Supplementary material for: Can Machine Learning Predict Metastatic Sites in Pancreatic Ductal Adenocarcinoma? A Radiomic Analysis
Source: J Imaging Inform Med. 2025 Aug 4;39(2):1536–46. doi: 10.1007/s10278-025-01627-y (PMC13103170; doi:10.1007/s10278-025-01627-y)
Supplement: Supplementary file 1 — Supplementary file1 (DOCX 16 KB) [file 10278_2025_1627_MOESM1_ESM.docx]

Table S1. Lists of all 42 retained features with ICC > 0.9, including their categories (GLCM, GLRLM, GLSZM, GLDM, First Order, and Shape-based), ICC values, and brief descriptions of each feature's meaning. This table provides comprehensive information about all reproducible features used in our initial analysis before LASSO feature selection

| **Feature Name** | **Category** | **ICC Value** | **Description** |
| --- | --- | --- | --- |
| **First Order Statistics** |  |  |  |
| Mean | First Order | >0.9 | Average gray level intensity within the ROI |
| Median | First Order | >0.9 | Median gray level intensity value |
| 10th Percentile | First Order | >0.9 | 10th percentile of gray level distribution |
| 90th Percentile | First Order | >0.9 | 90th percentile of gray level distribution |
| Interquartile Range | First Order | >0.9 | Difference between 75th and 25th percentiles |
| Skewness | First Order | >0.9 | Asymmetry of the gray level distribution |
| Kurtosis | First Order | >0.9 | Peakedness of the gray level distribution |
| Uniformity | First Order | >0.9 | Sum of squares of each intensity value |
| Energy | First Order | >0.9 | Magnitude of voxel values in an image |
| **Shape-based Features** |  |  |  |
| Volume | Shape | >0.9 | Volume of the region of interest in mm³ |
| Surface Area | Shape | >0.9 | Surface area of the region in mm² |
| Sphericity | Shape | >0.9 | Measure of how spherical the ROI is |
| Maximum 3D Diameter | Shape | >0.9 | Largest pairwise Euclidean distance |
| **Gray Level Co-occurrence Matrix (GLCM)** |  |  |  |
| Autocorrelation | GLCM | >0.9 | Measure of the fineness and coarseness of texture |
| Joint Average | GLCM | >0.9 | Average gray level intensity of the distribution |
| Cluster Prominence | GLCM | >0.9 | Measure of the skewness and asymmetry of the GLCM |
| Cluster Shade | GLCM | >0.9 | Measure of the skewness of the GLCM |
| Contrast | GLCM | >0.9 | Local intensity variation |
| Correlation | GLCM | >0.9 | Linear dependency of gray levels on neighboring pixels |
| Difference Average | GLCM | >0.9 | Mean of the diagonal probability distribution |
| Difference Variance | GLCM | >0.9 | Variance of the diagonal probability distribution |
| Joint Energy | GLCM | >0.9 | Measure of homogeneous patterns in the image |
| Joint Entropy | GLCM | >0.9 | Measure of the randomness/variability in neighborhood intensity values |
| **Gray Level Run Length Matrix (GLRLM)** |  |  |  |
| Short Run Emphasis | GLRLM | >0.9 | Distribution of short runs |
| Long Run Emphasis | GLRLM | >0.9 | Distribution of long runs |
| Gray Level Non-Uniformity | GLRLM | >0.9 | Variability of gray-level intensity values |
| Run Length Non-Uniformity | GLRLM | >0.9 | Variability of run lengths |
| Run Percentage | GLRLM | >0.9 | Fraction of realized runs and maximum possible runs |
| Low Gray Level Run Emphasis | GLRLM | >0.9 | Distribution of low gray-level values |
| High Gray Level Run Emphasis | GLRLM | >0.9 | Distribution of high gray-level values |
| Short Run Low Gray Level Emphasis | GLRLM | >0.9 | Joint distribution of shorter runs with lower gray-level values |
| **Gray Level Size Zone Matrix (GLSZM)** |  |  |  |
| Small Area Emphasis | GLSZM | >0.9 | Distribution of small size zones |
| Large Area Emphasis | GLSZM | >0.9 | Distribution of large size zones |
| Gray Level Non-Uniformity | GLSZM | >0.9 | Variability of gray-level intensity values |
| Zone Percentage | GLSZM | >0.9 | Fraction of voxels in zones |
| Low Gray Level Zone Emphasis | GLSZM | >0.9 | Distribution of low gray-level size zones |
| High Gray Level Zone Emphasis | GLSZM | >0.9 | Distribution of high gray-level size zones |
| **Gray Level Dependence Matrix (GLDM)** |  |  |  |
| Small Dependence Emphasis | GLDM | >0.9 | Distribution of small dependencies |
| Large Dependence Emphasis | GLDM | >0.9 | Distribution of large dependencies |
| Gray Level Non-Uniformity | GLDM | >0.9 | Similarity of gray-level intensity values |
| Dependence Non-Uniformity | GLDM | >0.9 | Similarity of dependence throughout the image |
| Dependence Percentage | GLDM | >0.9 | Fraction of voxels in the image that neighbor the same intensity value |
